# Do fine needle aspirate cytomorphological features correlate with positron emission tomography findings of metastatic non‐small cell lung carcinoma in lymph nodes?

**DOI:** 10.1002/cam4.5629

**Published:** 2023-01-23

**Authors:** Kianoosh Keyhanian, Harmanjatinder S. Sekhon

**Affiliations:** ^1^ Eastern Ontario Regional Laboratory Association, Department of Pathology and Laboratory Medicine University of Ottawa Ottawa Ontario Canada

**Keywords:** adenocarcinoma, cytology, fine needle biopsy, non‐small‐cell lung carcinoma, positron emission tomography, squamous cell carcinoma

## Abstract

**Background:**

Our objective was to correlate cytomorphological features of metastatic non‐small cell lung carcinoma (mNSCLC) with maximal standardized uptake value (mSUV) of positron emission tomography (PET) in Lymph nodes (LNs).

**Methods:**

Positive cytology slides of 114 LNs were reviewed from 100 patients with mNSCLC who had undergone PET study. Student's *t*‐test was used for statistical comparisons.

**Results:**

Mean patients' age: 68.5, 54% male. LNs locations were: mediastinum: 99, lung hilum: 13, peribronchial: 1, axilla: 1. Final diagnoses were: Adenocarcinoma: 86, squamous cell carcinoma: 28 LNs. Within the adenocarcinoma subgroup, histological patterns correlate with mSUV. Acinar and papillary patterns were associated with significantly lower mSUVs (mean ± standard error (SE): 7.9 ± 0.9 and 9.2 ± 0.8, respectively) than solid pattern (13.0 ± 1.2; *p* values: 0.001 and 0.009, respectively). Similar difference exists between patterns associated with low‐ and high‐grade adenocarcinoma (Mean ± SE: 9.2 ± 0.8 and 12.0 ± 1.0, respectively. *p* value: 0.02). Interestingly, micropapillary pattern was associated with the lowest mSUV amongst all patterns (Mean ± SE: 5.4 ± 1.1). Other features that correlated with higher mSUV were necrosis, moderate/severe nuclear atypia, lower lymphoid tissue yield, and contralateral LN involvement.

**Conclusions:**

In LNs with mNSCLC, certain cytomorphological features are associated with higher mSUV. Micropapillary, a pattern considered as high‐grade, is associated with lower SUV values; hence, a lower SUV threshold may raise concern for metastasis. Although high SUV is associated with LN metastasis, lower SUV levels in certain adenocarcinomas suggest correlation with clinical and morphological characteristics could be valuable in tailoring therapeutic management.

## Graphical abstract

In lymph node cytology specimens from non‐small cell lung carcinoma, certain cytopathological features including architectural pattern, grade, presence of necrosis, and moderate/severe nuclear atypia are associated with higher maximal standard uptake value of positron emission tomography.

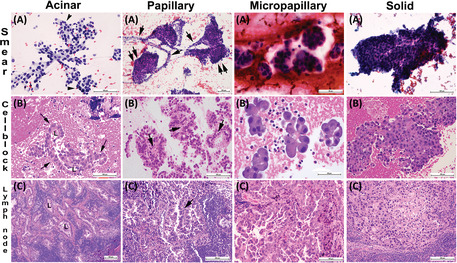

## 1 INTRODUCTION

World‐wide, lung cancer is one of the leading causes of cancer incidence, accounting for more than 2 million cases diagnosed globally in 2018.^1^ In Canada, lung cancer is the most commonly diagnosed cancer.^2^ Due to lack of wide‐spread tools and guidelines for lung cancer screening, approximately half of the patients present with advanced stage lung cancer at the time of diagnosis, contributing to the low overall survival rate.^2^ Lung cancer is also the leading cause of cancer mortality worldwide, accounting for 18.4% of cancer deaths globally.^1^ Non‐small cell lung cancer (NSCLC) accounts for about 80% of the lung cancer cases in Canada as well as in other countries.^2^, ^3^

Optimal management of NSCLC is largely dependent on accurate lymph node (LN) staging. Patients with no LN spread (N0), or intrapulmonary or ipsilateral hilar involvement (N1) are considered for surgical resection. On the other hand, N2 disease defined as metastasis to ipsilateral mediastinal LNs and/or subcarinal LNs, is treated with chemoradiation with or without surgery (controversial). Surgery is believed to have a limited role in patients with bulky stage III tumors (including N3), which often receive chemotherapy and radiation therapy.^4^

Glucose metabolism imaging in vivo using ^18^F‐ fluorodeoxyglucose (FDG) as a tracer, also known as positron emission tomography (PET), is used for mapping cancer spread within the body. Glucose metabolism and hence FDG uptake is elevated in most NSCLCs, making PET a valuable noninvasive option for accurate cancer staging.^5^

Standard uptake value (SUV) of FDG in the lung tumors has been the subject of multiple investigations. Comparing the disease outcome, some studies concluded that higher SUV of the primary tumor is correlated with shorter survival after treatment.^5^, ^6^, ^7^, ^8^, ^9^ Higher tumoral SUV max is also shown to be a risk factor for LN metastasis.^10^

Following PET/CT scan, obtaining tissue for histological assessment via less invasive endobronchial ultrasound guided transbronchial needle aspiration (EBUS‐TBNA) and/or core biopsy or more invasive mediastinoscopic biopsy is deemed essential for confirmation of the diagnosis.^11^ This is largely due to a relatively high rate of false positive PET/CT findings. EBUS‐TBNA is a minimally invasive procedure with similar sensitivity and higher specificity and accuracy comparing to PET and CT scan.^12^ Pre‐procedural CT or PET for selection of patients with suspected LN metastasis improves the sensitivity of EBUS‐TBNA.^13^ Therefore, in many institutions PET and/or CT scan followed by EBUS‐TBNA of the suspicious LNs is standard of care for pre‐operative staging of lung cancer.

Although the use of PET followed by EBUS‐TBNA is an established method of LN staging in NSCLC, architectural and cytological morphology of metastatic lung carcinoma has not yet been evaluated in association with corresponding PET findings. The objective of this study is to explore correlation of cytomorphological features of metastatic non‐small cell lung carcinoma (mNSCLC) with maximal standardized uptake value (mSUV) of PET scan in intrathoracic LNs.

## 2 MATERIALS AND METHODS

This study was approved by the institutional research ethics board of The Ottawa Hospital (protocol # 20180631‐01H). The Eastern Ontario Regional Laboratory Association (EORLA) Anatomic Pathology information System was searched for EBUS‐TBNA cytology cases of metastatic LNs (from 2016 to 2017). One‐hundred and fourteen (114) positive LNs were enrolled from 100 cases with NSCLC. Positive EBUS‐TBNA cytology slides were retrieved and reviewed. LNs with the final cytological diagnoses of metastatic adenocarcinoma (AdCa) and squamous cell carcinoma (SqCC) were included in the study if the PET characteristics of the corresponding LNs were available. When the cytomorphology was not definitive, immunohistochemistry (P40, TTF1, and CK7) was used to assist in differentiating between AdCa and SqCC. Small cell carcinoma, metastatic carcinoma of non‐lung origin, and LNs with cytological diagnoses of “cytologic atypia” or “positive for malignancy” (where there was insufficient material to ascertain a diagnosis of NSCLC) were not included in this study.

**FIGURE 1:**
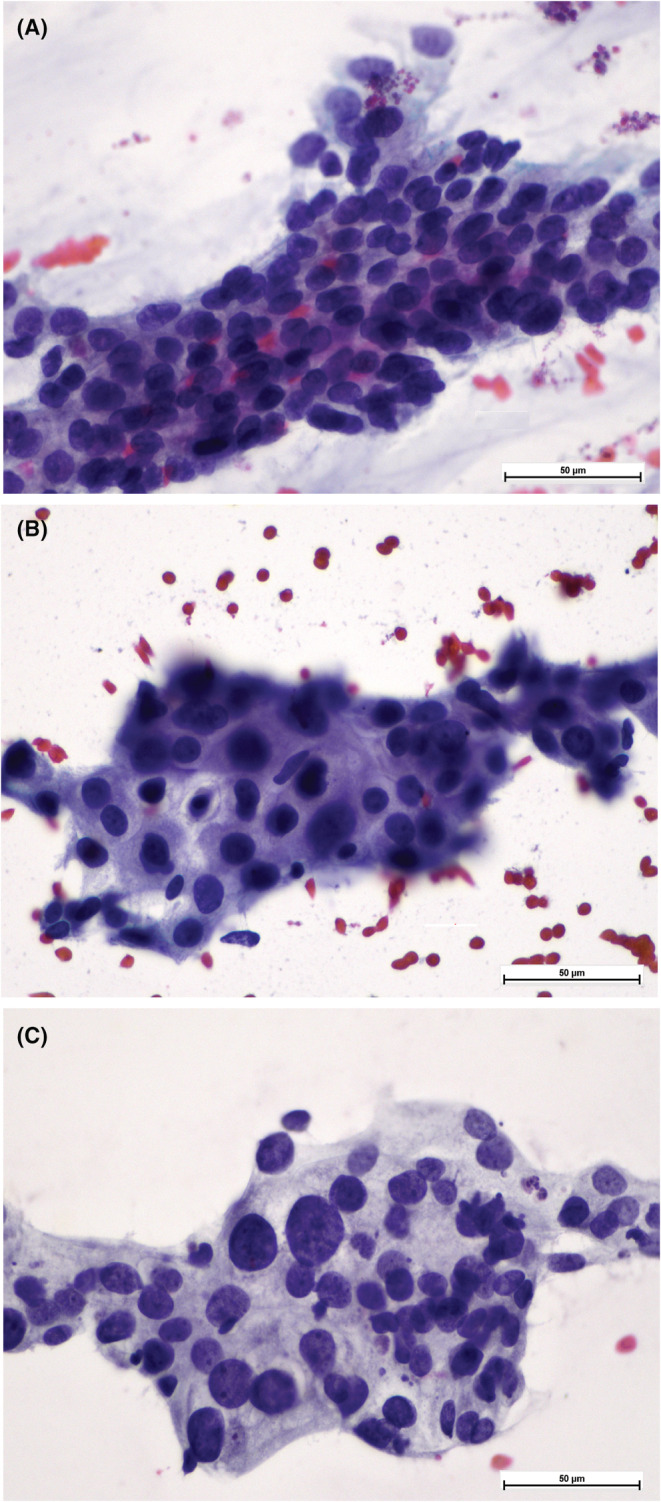
Nuclear atypia assigned as mild (A), moderate (B), and severe (C) in smears, Pap‐stained, 400×. The nuclear atypia was considered moderate if there was nuclear size variation of more than two times (B) and severe if there were significant number of nuclei with nuclear size variation of three times (C).

**FIGURE 2:**
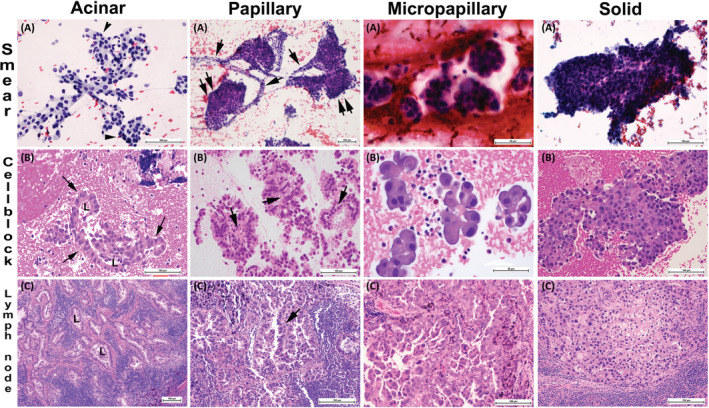
The microphotographs show features associated with acinar, papillary, micropapillary, and solid architectural patterns of adenocarcinoma in endobronchial ultrasound guided fine needle aspirate (EBUS‐FNA) specimens. (A) represent fine needle aspirate (FNA) Pap‐stained smears, (B) H&E‐stained section of cell blocks, and (C) histological H&E‐stained sections of resected lymph nodes. Acinar panel: (A). The microphotograph shows mild to moderately atypical cells arranged in mainly monolayer sheets containing mildly pleomorphic nuclei and vesicular cytoplasm (200×); (B) section of cell block depicts glandular cell arrangement characterized by smooth outer border of gland and basally placed nuclei toward the outer border (single arrow). The luminal border (L) is slightly irregular facing the cytoplasm of columnar or cuboidal cells (200×); (C) section of lymph node with acinar arrangement of metastatic carcinoma (100×). Papillary panel: (A) Lymph node smear shows mild to moderately atypical cells arranged in papillary configuration with stranding capillaries (single arrows) interconnecting the cell groups that have smooth arrangement of cells as apical caps (double arrows, 100×); (B) cell block show typical fibrovascular cores with outer columnar cells arrangement (200×). Similarly, the cell groups that do not have defined fibrovascular cores (single arrow) exhibit the reverse of the acinar arrangement with smooth inner outline (single arrow) and basally located nuclei whereas the outer border is irregular facing the cytoplasm of columnar cells containing cytoplasmic vacuoles. (C) lymph node with metastatic carcinoma of papillary pattern (200×). Micropapillary Panel: (A) Smear shows small three‐dimensional groups of moderate to markedly atypical cells arranged in nests/balls with scalloped outer borders and no apparent fibrovascular core (florets). The cells contain moderately pleomorphic nuclei and moderate to ample microvascular or vacuolated eosinophilic cytoplasm (400×); (B) cell block shows cells with floret‐like arrangement with scalloped outer borders and no obvious fibrovascular cores (400×); and (C) section of lymph node with metastatic carcinoma of micropapillary pattern (200×). Solid panel: (A) Smear with large sheets of over‐crowded markedly atypical cells with no apparent arrangement. The outer borders are rough and uneven (200×); (B) cell block exhibiting markedly pleomorphic cells in large sheets with no other architectural pattern. Some cells have cytoplasmic vacuoles or clearing (200×); (C) section of lymph node with solid pattern similar to what is seen in the cell block (200×).

When reviewing tumor cytomorphology, the nuclear atypia was considered moderate if there was nuclear size variation of more than two times and severe if there were significant number of nuclei with nuclear size variation of three times (Figure 1). The cytomorphological features of various AdCa patterns was adopted from the previous study in our institution, which is based on the corresponding histological AdCa pattern defined by WHO Classification of Tumors of the Lung, Pleura, Thymus and Heart^14^, ^15^ (Figure 2). The necrosis assessment was done if necrotic tumor were seen either in the smears or in the sections of cell block(s).

The tumor yield was assessed based on approximate number of tumor cells present collectively in the smears and the sections of cell block and was classified into four categories: category 1 corresponding to <100 tumor cells, 2 corresponding to 100–300 cells, 3 corresponding to 300–500, and 4 corresponding to >500 cells. Similarly, the LN elements (lymphocytes, histiocytes or macrophages) yield was also classified in four categories: category 1 corresponding to the small aggregates (less than 200 cells) collectively on the smears and in the sections of cell blocks; category 2 as large aggregates of lymphocytes and histiocytes (more than 200 to about 500); category 3 as pools of lymphocytes or histiocytes (500–1000 cells) and category 4 as large pools of abundant cells (>1000 cells). Assessment of tumor yield and lymphoid yield was performed by authors at the time of cytology review. This categorization is routinely used in our institution for reporting LN adequacy in EBUS‐TBNA specimens.

Student's *t*‐test was used for statistical comparison, performed on Microsoft Excel for Mac version 16.68. Pearson correlation coefficient was calculated, using MATLAB, to determine the correlation of LN sizes with their corresponding SUV as well as the correlation of primary tumor SUV with corresponding LN SUV. A *p*‐value <0.05 was considered statistically significant.

## 3 RESULTS

### 3.1 Patient population

In total, 114 LNs were examined form 100 patients. Mean patients' age was 68.5 years old (69.2 for male and 67.1 for female patients). About 54% were male and 46% were females. LNs locations were as follows: mediastinal: 99 (44 subcarinal, 3 precarinal, 52 para‐tracheal), hilar: 13, Peribronchial: 1, axillary: 1. Overall, 86 LNs contained metastatic AdCa (75%) and 28 contained metastatic SqCC (25%; Table 1). No specimen was excluded from this study based on the quantity of lymphoid elements.

**TABLE 1:** Patient demographics and primary lung tumor characteristics

	All patients	Hilar or Peribronchial LNs	Mediastinal LNs	Contralateral LNs
Age	68.5	68	68.5	66.9
% Male	54	58	49	70
Tumor size (cm)	4.3	2.7	4.5	4.9
Tumor location				
LUL/LLL	12^a^/9	1/3	5/4	5/2
RUL/RML/RLL	36/7/31	4/1/3	32/6/26	0/0/2
Histological subtype				
AdCa	86	10	70	6
SqCC	28	3	22	3
Primary tumor SUV	13.5	10.0	13.8	14.9

Abbreviations: AdCa, Adenocarcinoma; LLL, Left lower lobe; LN, Lymph node; LUL, Left upper lobe; RLL, Right lower lobe; RML, Right middle lobe; RUL, Right upper lobe; SqCC, Squamous cell carcinoma; SUV, standard uptake value.

^a^ There was one additional axillary lymph node.

### 3.2 Cytomorphology and PET mSUV correlation

**FIGURE 3:**
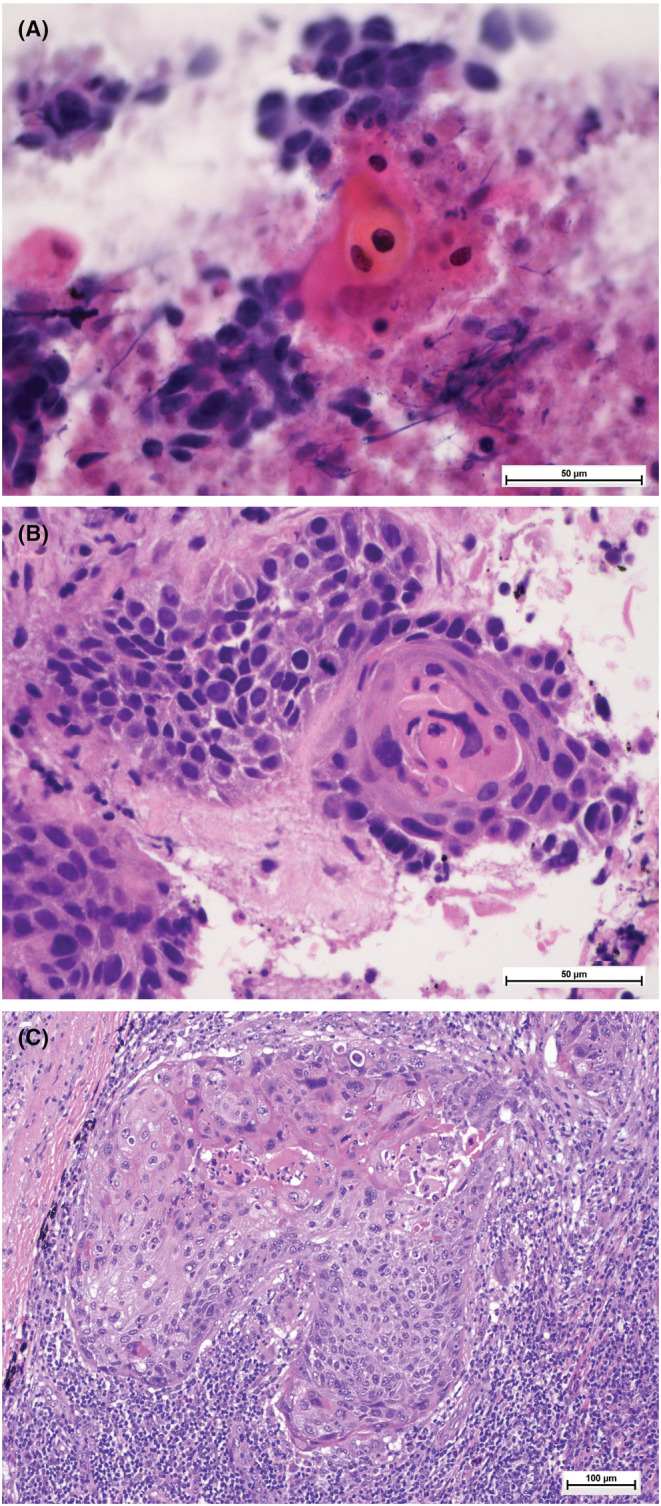
Metastatic squamous cell carcinoma. (A) Smear, Pap‐stained, 200× depicting nests of atypical cells with pleomorphic and hyperchromatic nuclei, eosinophilic cytoplasm, and keratinization (B) Cell block, H&E stained, 200× (C) H&E stained histological section of lymph node with metastatic squamous cell carcinoma.

**FIGURE 4:**
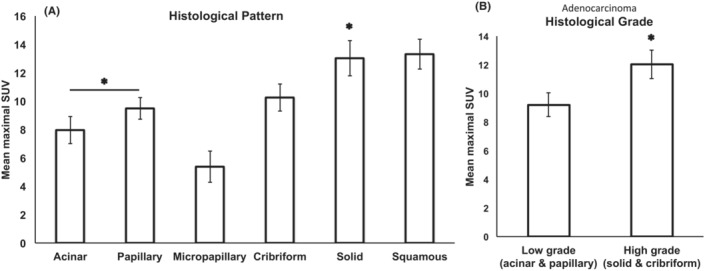
Architectural patterns in LN cytology specimens of NSCLC and correlation with SUV value. (A) Acinar and papillary (mean ± SE: 7.9 ± 0.9 with *n* = 17 and 9.2 ± 0.8 with *n* = 31, respectively) architectural patterns are associated with significantly lower mSUVs than solid pattern (Mean ± SE: 13.0 ± 1.2, *n* = 14; *p* values: 0.001 and 0.009, respectively). Micropapillary pattern was associated with the lowest mSUV amongst all patterns (Mean ± SE: 5.4 ± 1.1 with *n* = 8). The mean mSUV of micropapillary (Mean ± SE: 5.4 ± 1.1) was significantly lower than the low‐grade patterns (acinar and papillary; *p* value: 0.035) as well as the high‐grade patterns (cribriform and solid; *p* value: 0.000). (B) On average, patterns associated with low‐grade adenocarcinoma that is, acinar and papillary have lower mSUVs versus high grade patterns that is, cribriform and solid (Mean ± SE: 9.2 ± 0.8 and 12.0 ± 1.0, respectively. *p* value: 0.02). SUV: standard uptake value.

Metastatic AdCa in LNs (Figure 2) presented with mean mSUV of 9.4 (standard error (SE): 0.5) compared to metastatic SqCC 13.3 (SE: 1.0; Figure 3). We then assessed metastatic AdCa cytomorphological features corresponding to the histological AdCa subtypes including acinar, papillary, micropapillary, cribriform and solid^14^ and assigned each case with the predominant pattern (Figure 2). Our results indicated that in metastatic AdCas, predominant cytological pattern of growth corresponding to histological subtypes correlates with mSUV. AdCa with acinar and papillary patterns are associated with significantly lower mSUVs (mean ± SE: 7.9 ± 0.9 with *n* = 17 and 9.2 ± 0.8 with *n* = 31, respectively) compared to solid pattern (13.0 ± 1.2, *n* = 14; *p* values 0.001 and 0.009, respectively). Similar difference in mSUV exists when combining the categories corresponding to low‐grade AdCa that is, acinar and papillary versus high grade patterns that is, cribriform and solid (9.2 ± 0.8 and 12.0 ± 1.0, respectively. *p* value: 0.02; Figure 4A,B).

Interestingly, although micropapillary AdCa is considered a high‐grade pattern, it was associated with the lowest mSUV amongst all AdCa patterns (Mean ± SE: 5.4 ± 1.1, *n* = 8). The mean mSUV of micropapillary was significantly lower than the low‐grade patterns (acinar and papillary; *p* value <0.03) as well as the high‐grade patterns (cribriform and solid; *p* value <0.0001). The mSUV of cases with micropapillary predominant pattern ranged from 2.9 to 12.7. Notably, 50% (4/8) of these cases had an SUV value ≤4.

It is worth noting that SqCC group showed an average mSUV similar to the solid‐pattern AdCas (mean ± SE: 13.3 ± 1.0, *n* = 14) that was significantly higher than other metastatic AdCa subcategories (Figure 4A).

In order to further explore the relationship of low SUV in metastatic AdCa with micropapillary cytomorphology, we assessed the LN positivity in a cohort of 40 micropapillary predominant and 110 papillary predominant primary lung AdCas resected in our institution. We found that 45% (18/40) of micropapillary predominant AdCa cases were node positive on resection, in comparison to only 18% (20/110) of papillary predominant AdCas (*p* = 0.003). The mean SUV of the involved LNs was 3.9 (*n* = 6) for the micropapillary‐predominant AdCa cases compared to mean LN mSUV of 5.4 (*n* = 6) for papillary‐predominant AdCa cases (*p* = 0.14).

We then explored the architectural pattern in metastatic LNs of resected micropapillary predominant primary lung AdCas. There were six resected lung AdCas with nodal involvement and predominant micropapillary pattern in our institution since 2014. In all these cases, the predominant pattern of metastasis in the LNs was in fact also micropapillary.

**FIGURE 5:**
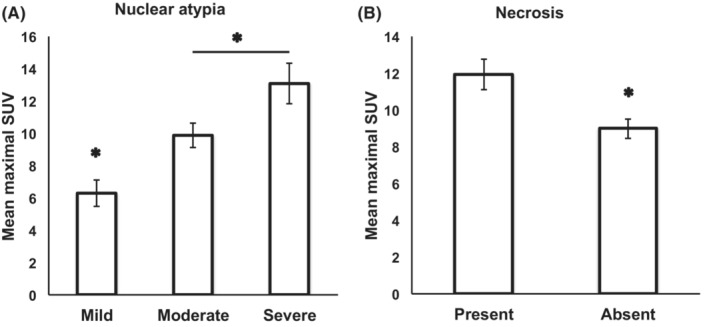
(A) LNs with metastases harboring moderate and severe nuclear atypia (mean ± SE: 13.1 ± 1.3) were associated with significantly higher mSUVs compared to metastatic tumors with mild nuclear atypia (mean ± SE: 6.2 ± 0.8, *p* value: 0.01). (B) Tumors featuring necrosis were associated with higher SUVs compared to the ones without necrosis (mean ± SE: 11.9 ± 0.8 vs. 9.0 ± 0.5, *p* value: 0.001). SUV: standard uptake value.

**FIGURE 6:**
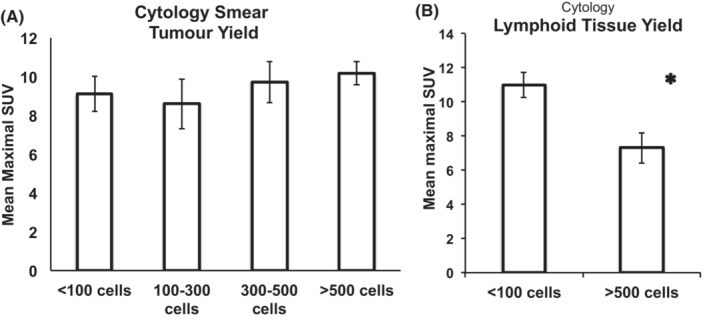
(A) There was no significant difference between tumor yield on the smear and the mSUV value. Number of cases in each category is: <100 cells: 12 cases (10 AdCa, 2 SqCC), 100–300 cells: 10 cases (9 AdCa, 1 SqCC), 300–500 cells: 20 cases (17 AdCa, 3 SqCC), >500: 67 cases (45 AdCa, 22 SqCC). (B) MSUV was significantly higher in LNs with <100 lymphoid cell yield versus the ones with >500 lymphoid cell yield (mean ± SE: 11.0 ± 0.7 vs. 7.3 ± 0.9. *p* value: 0.04). SUV: standard uptake value.

**FIGURE 7:**
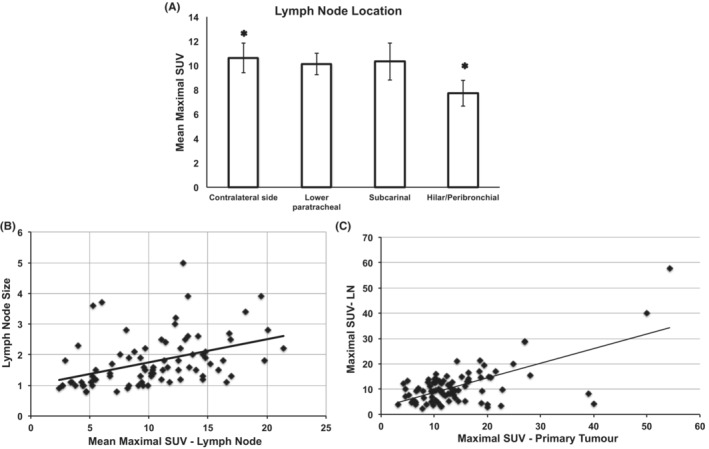
(A) Contralateral mediastinal and hilar LN involvement is associated with significantly higher mSUV values than average ipsilateral hilar/bronchial LNs (mean ± SE: 10.6 ± 1.2 vs. 7.7 ± 1.0, *p* value: 0.04). (B) Using short axis dimension of the LN, there was a weak correlation between the LN size and SUV with correlation coefficient of 0.42 and *p* value of 0.000 (*N*: 83). (C) mSUV of LNs was moderately correlated with that of the primary tumors (*N*: 89, correlation coefficient: 0.0.65 and *p* value: 0.000). LN: Lymph node. SUV: standard uptake value.

The following results (presented in Figures 5, 6, 7) are performed on pulled AdCa and SqCC cases. Apart from the architectural pattern of growth, we also compared other features such as nuclear atypia and presence of necrosis in relation to the LN mSUV. LNs with metastases harboring moderate and severe nuclear atypia were associated with significantly higher mSUVs (mean ± SE: 13.1 ± 1.3) compared to metastatic tumors with mild nuclear atypia (mean ± SE: 6.2 ± 0.8, *p* value: 0.01; Figure 5A). Also, tumors featuring necrosis were associated with higher SUVs compared to the ones without necrosis (mean ± SE: 11.9 ± 0.8 vs. 9.0 ± 0.5, *p* value: 0.001; Figure 5B). Within cases with necrosis, 42% were SqCC (19/45).

### 3.3 Cellular yield

We compared the mSUV amongst the cases with different tumor smear yield as well as lymphoid yield. Since the number of smear tumor cells is highly variable, we categorized the cellularity to one through four, with category 1 corresponding to <100 tumor cells on the smear, 2 corresponding to 100–300 cells, 3 corresponding to 300–500, and 4 corresponding to >500 cells. Based on our data, there was no significant difference between these four categories and the mSUV value (Figure 6A). On the other hand, comparing the lymphoid tissue yield revealed significantly higher SUV in LNs with <100 lymphoid cell yield versus the ones with >500 lymphoid cell yield (mean ± SE: 11.0 ± 0.7 vs. 7.3 ± 0.9, *p* value: 0.04; Figure 6B).

### 3.4 Lymph node location, size, and correlation with primary tumor

Nodal staging of the lung carcinomas follows the anatomical lymphoid drainage route, with ipsilateral peribronchial and hilar LN involvement considered as N1 disease, ipsilateral mediastinal and subcarinal involvement as N2 and contralateral mediastinal or hilar involvement as N3 disease. Our data demonstrates that there is a trend for a higher mSUV as the nodal staging progresses. Also, contralateral mediastinal and hilar LN involvement is associated with significantly higher mSUV values than average ipsilateral hilar/bronchial lymph nodes (mean ± SE: 10.6 ± 1.2 vs. 7.7 ± 1.0, *p* value: 0.04; Figure 7A).

We then calculated the correlation between LN size and mean maximal SUV. Using short axis dimension of the LN, there was a weak correlation between the LN size and SUV with correlation coefficient of 0.42 and *p* value of 0.000 (Figure 7B).

On a separate note, in our cohort, mSUV of LNs was moderately correlated with that of the primary tumors (*N*: 89, correlation coefficient: 0.65 and *p* value: 0.000; Figure 7C).

## 4 DISCUSSION

In this study we evaluated the morphology of metastatic NSCLC in thoracic LNs in correlation with their PET findings. Our data suggests that in LN cytology specimens, certain cytopathological features including histological pattern, presence of necrosis and moderate/severe nuclear atypia are associated with higher mSUV, consistent with prior studies examining primary lung lesions.^5^, ^16^

In primary lung cancer, histological subtypes have been correlated by other studies with SUV. Vesselle et al. showed that adenocarcinoma in situ has the lowest mean SUV than other lung cancer histological subtypes.^5^ They also reported that AdCa has lower average uptake of FDG than SqCC and large cell undifferentiated carcinoma. Also, poorly differentiated carcinomas were shown to be more FDG avid.^5^ Moreover, FDG SUV has been demonstrated to have a significant correlation to Ki67 proliferation index in the primary lung tumors.^5^, ^17^ Our findings are complementary to the previous studies adding that aggressive biological features (patterns associated with higher grade, nuclear atypia, and necrosis) of the tumor in cytology of the LN correlate with higher SUV.

An interesting and important finding of our study was the fact that LNs with predominant micropapillary growth pattern on cytology showed the lowest mSUV amongst other patterns. Micropapillary‐predominant AdCas metastasize early and impart aggressive clinical behavior and poor prognosis.^15^ One explanation for low SUV in these tumors is their low cellular proliferative rate^18^ compared to the other aggressive AdCa subtypes. This finding, if validated by subsequent studies, has a significant clinical implication in the management of cases with micropapillary pattern on cytology and borderline SUV numbers on PET scan. Given the high rate of LN positivity in micropapillary predominant lung AdCas, we believe that the presence of this pattern in a LN biopsy or cytology sample is a reportable finding. Describing the identifiable patterns in small biopsies/cytology specimens is previously recommended by International Association for the Study of Lung Cancer/American Thoracic Society/European Respiratory Society.^19^ If feasibility and reproducibility is confirmed by future studies, presence of micropapillary pattern has the potential to trigger consideration for a lower threshold of PET positivity for sampling or resection of the subsequent LNs.

The micropapillary and papillary patterns are rather easy to identify on the smears and/or on the sections of cell blocks as the papillary and micropapillary structures are easily dislodged and aspirated by fine needle compared to the other AdCa patterns. It was also interesting to note that the micropapillary pattern in the metastatic LNs in the resected specimens was also similar to that in the micropapillary predominant primary lung tumors examined in the archived cases. In fact, the node‐positive, micropapillary predominant primary lung AdCas in our institution demonstrated micropapillary to be the predominant pattern of LN metastasis. Given the low incidence of primary AdCas with predominant micropapillary pattern, one limitation of this study is a low number of metastatic LNs with micropapillary pattern. Therefore, an overall relationship between micropapillary pattern in the LN with primary pattern of lung primary remains to be investigated.

Although the tumor cellular yield did not correlate with an increase in SUV, lower lymphoid yield was associated with higher mSUV. One would expect that LNs with high mSUV levels likely have high tumor burden and lower proportion of normal LN components. Therefore, if the SUV levels are high along with increased LN size, negative EBUS result raises the possibility of inadequate sampling versus reactive LN. In case of a reactive LN leading to increased size, a higher yield of lymphoid elements is expected. A low lymphoid yield from a small PET negative LN may be acceptable; but not in an enlarged and/or PET positive LN. This common diagnostic challenge begs for a need of reporting adequacy in LN samples. The lymphoid tissue sampling categorization used in this study has been used for the past 5 years at our institution to report adequacy of LN sampling. This helps the clinicians and surgeons to assess whether or not the suspicious LNs were adequately sampled. It also serves as a tool to provide feedback for quality assurance and improvement. Therefore, in our opinion, the cytology report should routinely include a quantitative or semi‐quantitative statement about sampling adequacy including all lymphoid elements in the presence of PET positive or suspicious LNs.

One limitation of our study is lack of cytology‐histology correlation between the sampled LNs and the corresponding resected primary tumor and LNs. However, as mentioned in the results section, majority of the enrolled cases had mediastinal LN metastasis (out of 114 LNs, mediastinal: 99 (44 subcarinal, 3 precarinal, 52 para‐tracheal)), and were not followed by the resection of primary tumor. Therefore, adequate case numbers in different categories were not available for a meaningful correlation.

Modern practice of medicine mandates enhanced inter‐specialty communication and consultation for optimized patient care. Relaying the PET findings along with other imaging modalities to the cytopathologists as a part of clinical information can facilitate cytology specimen assessment and improves diagnostic precision. Our results show that micropapillary pattern of AdCa has relatively low SUV. Where possible the cytopathologists / morphologists should attempt to report patterns of lung AdCa to improve our understanding of lung AdCa patterns in relation to metabolic activity.

## AUTHOR CONTRIBUTIONS

**Kianoosh Keyhanian:** Conceptualization (equal); data curation (equal); formal analysis (equal); methodology (equal). **Harmanjatinder S. Sekhon:** Conceptualization (lead); data curation (supporting); formal analysis (supporting); methodology (equal).

## CONFLICT OF INTEREST

Authors declare no conflict of interest.

## ETHICAL APPROVAL

This research project was approved by Ottawa Health Science Network Research Ethics Board.

## Data Availability

The data that support the findings of this study are available from the authors upon request.
